# Supplementary material for: The motor neuron m6A repertoire governs neuronal homeostasis and FTO inhibition mitigates ALS symptom manifestation
Source: Nat Commun. 2025 Apr 30;16:4063. doi: 10.1038/s41467-025-59117-2 (PMC12043976; doi:10.1038/s41467-025-59117-2)
Supplement: Supplementary file 1 — Supplementary Information [file 41467_2025_59117_MOESM1_ESM.pdf]

Supplementary Fig.1

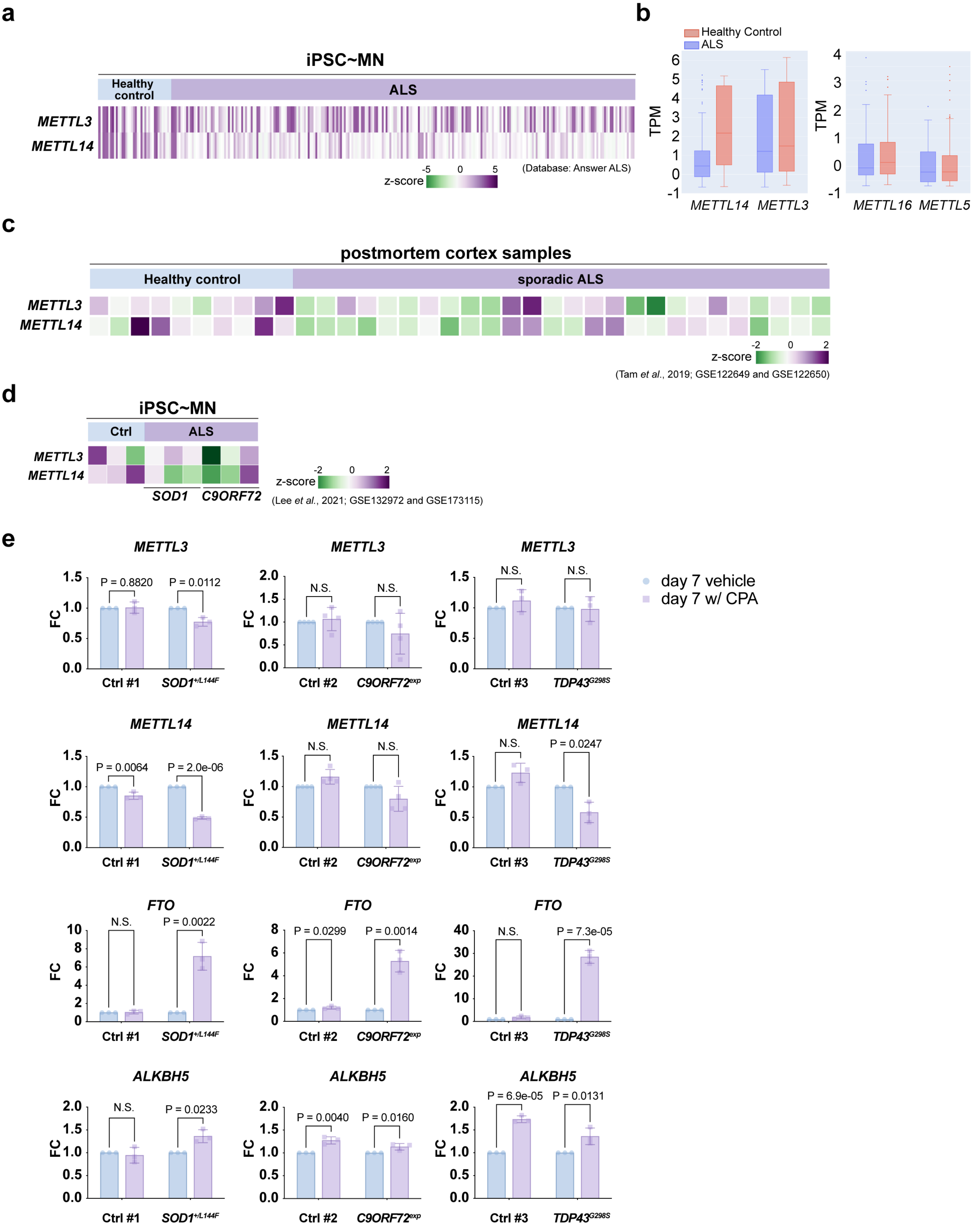

Supplementary Fig. 1: Expression of m^{6}A writers and erasers in human ALS iPSC-derived motor neurons. **a** and **b** Heatmaps (**a**) and corresponding box plots (**b**, left) of the m^{6}A 'writer' complex core components in the iPSC-derived motor neurons (iPSC~MNs) of randomly selected controls (n = 336) and ALS patients (both familial and sporadic, n = 855). Other m^{6}A modification enzymes, such as *METTL16* for ncRNAs or *METTL5* for rRNAs remain unchanged in ALS iPSC~MNs (**b**, right). Data were analyzed using the transcriptomic database from Answer ALS. Box plots indicate the interquartile range (IQR), with the central line revealing the median value and the vertical lines extending to the extreme values in the group. **c** Heatmaps of the m^{6}A 'writer' complex core components in the post-mortem cortex samples of randomly selected healthy controls (n = 10) and sporadic ALS patients (n = 26) of the GSE122650 database. **d** Heatmaps of the m^{6}A 'writer' complex core components in *SOD1* and *C9ORF72* iPSC~MNs and healthy controls (n = 3, respectively). Data was derived from the GSE132972 and GSE173115 databases. **e** qPCR analysis of m^{6}A writers (*METTL3* and *METTL14*) and erasers (*FTO* and *ALKBH5*) in human ALS iPSC~MNs upon degeneration induced by CPA treatment (see Methods for details). Note the general trend of downregulation for the m^{6}A writers and upregulation of erasers upon MN degeneration in the ALS-associated lines compared to their isogenic rescue controls. Data are presented as mean ± S.D., (n = 3, only n =4 in *METTL3* and *METTL14* qPCR from *C9ORF72^{exp}* ALS iPSC~MNs), with significant P values from two-tailed t-tests. N.S., non-significant. Source data are provided as a Source data file.

Supplementary Fig.2

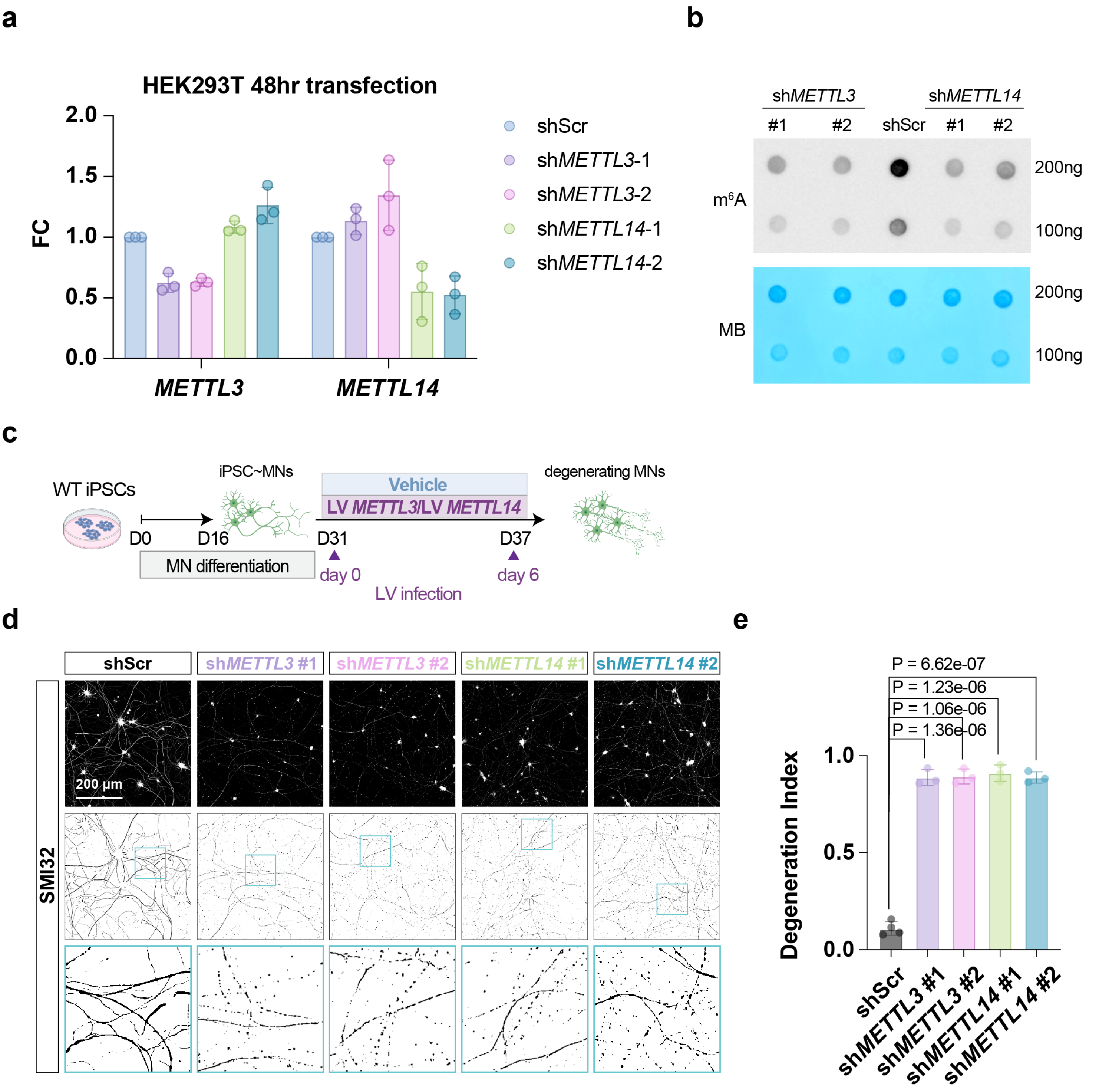

Supplementary Fig. 2: Knockdown efficiency test for sh*METTL3* and sh*METTL14.* a and **b** Knockdown of *METTL3* and *METTL14* in HEK293T cells by two individual shRNA clones led to downregulation of *METTL3* and *METTL14*, as determined by qPCR (**a**), *n* = 3 independent experiments with a sharp decline of m^6A mRNA levels, as assayed by m^6A dot blot and methylene blue staining (for loading controls) (**b)**. **c** Timeline of Lenti virus (LV)-mediated degeneration of MNs. Created in BioRender. Chen, J. (2025) https://biorender.com/3nizfu7. **d** and **h** Knockdown of *METTL3* and *METTL14* significantly increases the degeneration of WT iPSC-MNs. **d** The upper panel shows representative immunostainings of SMI32 from LV-infected WT iPSC-MNs. In the middle panel, a binarized image of neurons with cell bodies removed and neurite fragments. In the lower panel, an enlarged region of the binarized neurite image. Scale bar, 200 µm. **e** Quantification of the results from **d**. The degeneration index (DI) measures neurite fragmentation. It is calculated by dividing the total area covered by neurite fragments by the total neurite area, and values range from 0 (completely intact) to 1 (completely fragmented). Data are presented as mean ± S.D., shScr: *n* = 4, others: *n* = 3 independent experiments, significant P values from two-tailed *t*-tests. Source data are provided as a Source data file.

Supplementary Fig.3

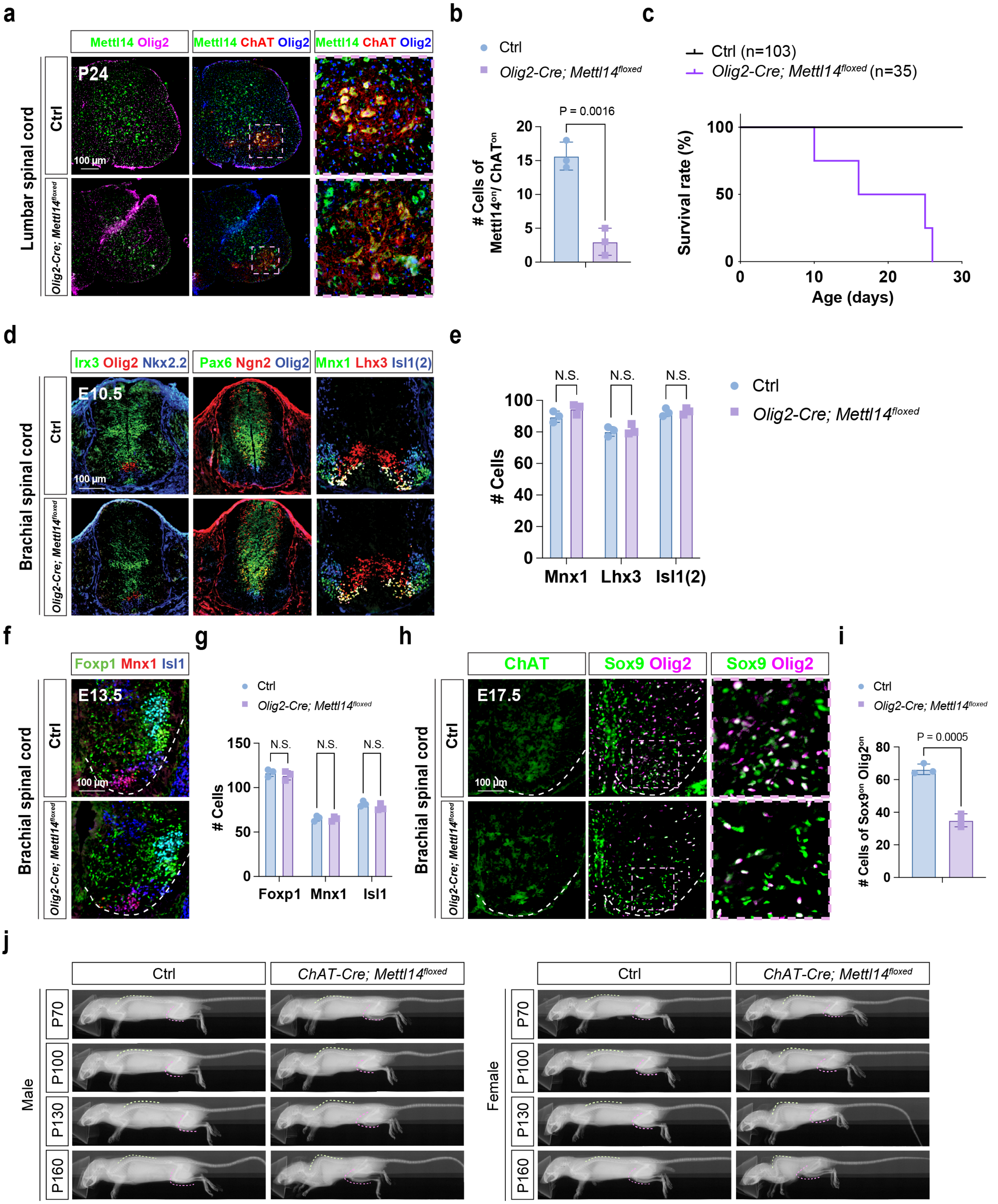

Supplementary Fig. 3: Phenotypic characterization of *Olig2-Cre; Mettl14^{floxed}* and *ChAT-Cre; Mettl14^{floxed}* mutant mice. a and **b** Immunostainings of Mettl14, Olig2, and ChAT in the spinal cord sections of P24 *Olig2-Cre; Mettl14^{floxed}* and littermate control mice **a**, together with respective quantification **b**. The ventral horn is framed by the dashed squares in the middle column and is zoomed out in the rightmost column. **c** Kaplan-Meier survival curves show that *Olig2-Cre; Mettl14^{floxed}* mice die postnatally (i.e., before P30) compared to littermate controls. **d** and **e** Specifications of neuronal progenitors (Irx3^{on}, Pax6^{on}/p0~p2, Olig2^{on}/pMN, and Nkx2.2^{on}/p3) and generic MNs (Isl1(2)^{on} or Mnx1^{on}) are not affected in *Olig2-Cre; Mettl14^{floxed}* spinal cords at E10.5. **f** and **g** Immunostaining for Foxp1, Mnx1, and Isl1 at E13.5 reveals comparable numbers of columnar MN subtypes between spinal cords of Ctrl and *Olig2-Cre; Mettl14^{floxed}* mice. **h** and **i** Expression of OPC (oligodendrocyte precursors) and astrocyte markers in E17.5 control and *Olig2-Cre; Mettl14^{floxed}* mice. The ventral horn is framed by the dashed squares in the middle column and is zoomed out in the rightmost column. Scale bars, 100 µm. **j** Roentgenograms of mice revealing obvious kyphosis in the *ChAT-Cre; Mettl14^{floxed}* mice. Purple dotted lines depict the hindlimb shape; green dotted lines depict the mouse spine (n = 3 mice). All data from **b**, **e**, **g**, and **I** are presented as mean ± S.D., n = 3 mice, with significant P values from two-tailed t-tests. N.S., non-significant. Source data are provided as a Source data file.

Supplementary Fig.4

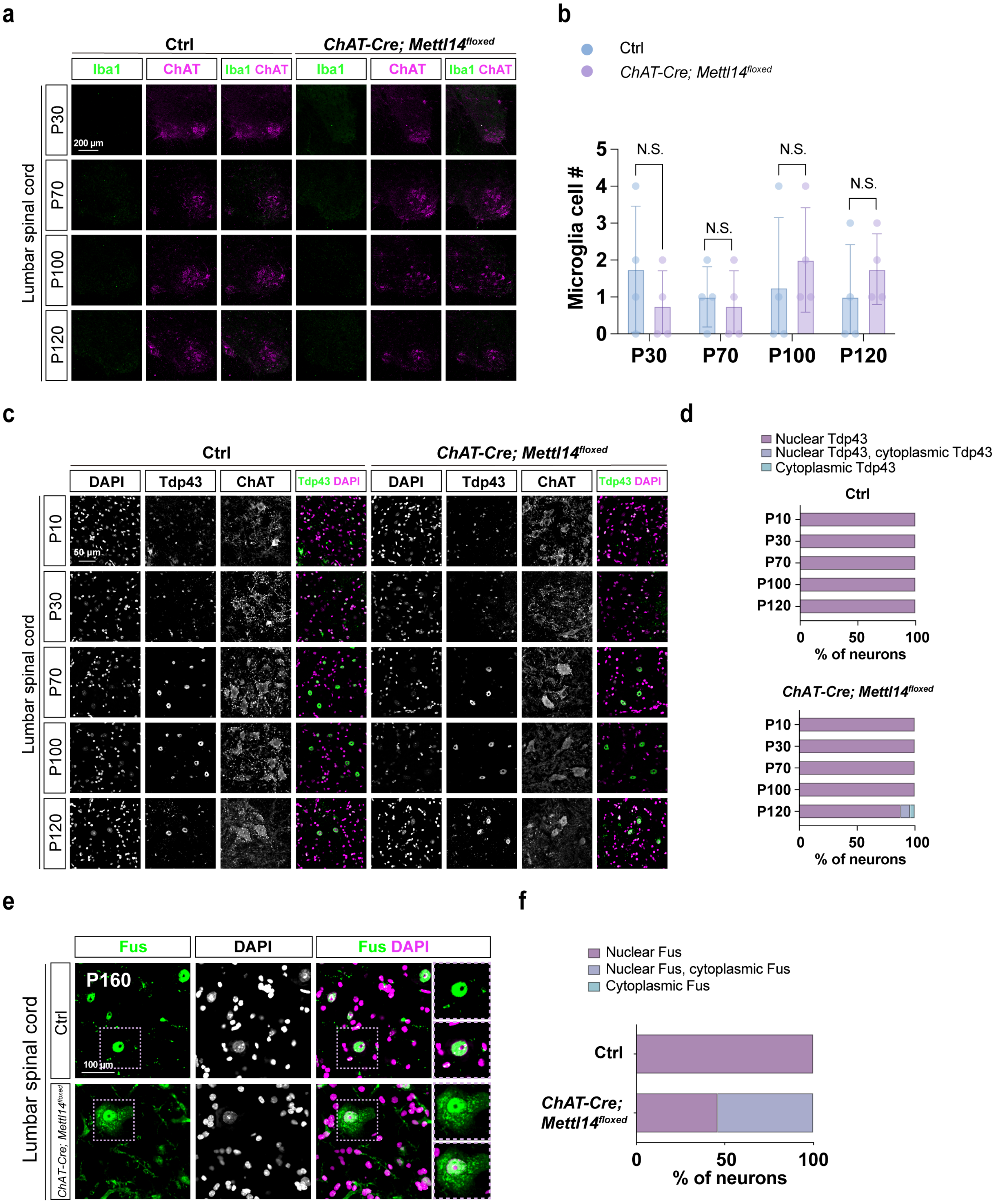

Supplementary Fig. 4: Profiling phenotypes of *ChAT-Cre; Mettl14^{floxed}* mice from postnatal to adult stages. Images illustrating immunostaining **a** and quantification **b** of lumbar Iba1^{on} numbers in the ventral region reveal no significant microglial activation in P30 ~ P120 *ChAT-Cre; Mettl14^{floxed}* mice compared with littermate controls. (*n* = 4 mice). Scale bars, 200 μm. **c** Tdp43 (green) is localized in the nucleus of the MNs in P10 ~ P100 *ChAT-Cre; Mettl14^{floxed}* mice. Scale bars, 50 μm. Respective quantification is presented in **d**. **e** RNA binding protein Fus is localized in the nucleus of the MNs of normal mice. In the *ChAT-Cre; Mettl14^{floxed}* mutant mice, numerous Fus inclusions exist in the cytoplasm. Scale bars, 100 μm. Respective quantification is presented in **f**. All data from **d** and **f** are presented as mean ± S.D., *n* = 3 mice, with significant P values from two-tailed *t*-tests. N.S., non-significant. Source data are provided as a Source data file.

Supplementary Fig.5

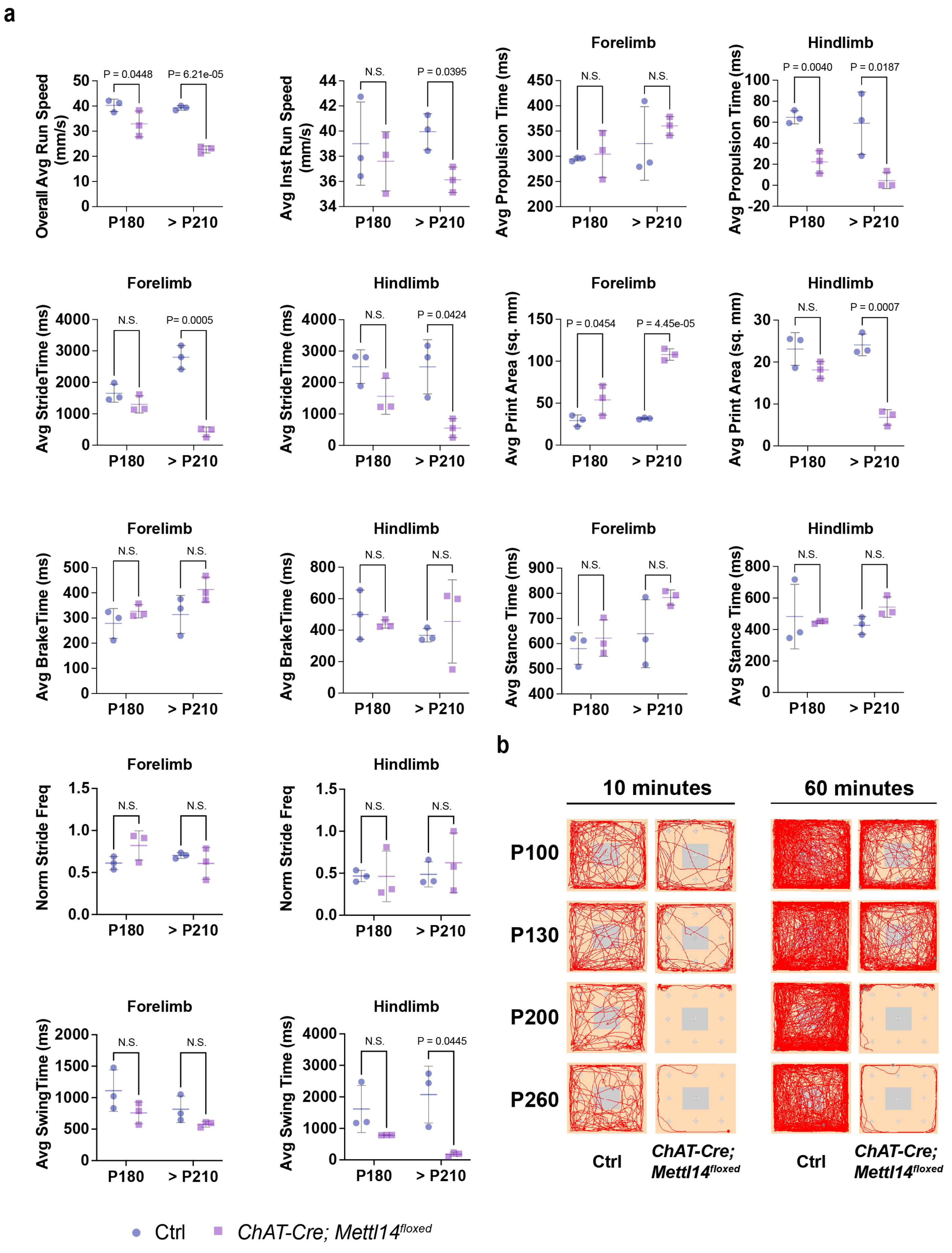

Supplementary Fig. 5: *ChAT-Cre; Mettl14^{floxed}* mice display overt motor deficits but no obvious defect in interneuron-mediated coordination. a Limb coordination and gait analysis were assayed by treadmill walking. *ChAT-Cre; Mettl14^{floxed}* mice display comparable temporal parameters of hindlimb stance, swing, brake, and propulsion time to controls, but their hindlimb walking ability is seriously compromised. Speed = 15 cm/sec (*n* = 3 mice). **b** *ChAT-Cre; Mettl14^{floxed}* mice display a gradual decrease of total distance traveled in the open field test. Data are presented as mean ± S.D., *n* = 6 mice, with significant P values from two-tailed *t*-tests. N.S., non-significant. Source data are provided as a Source data file.

Supplementary Fig.6

a

sample type	replicate 1	replicate 2	replicate 3
**No. of sequenced reads**	2324864	941873	1648826
**No. of mapped reads**	2063725	682478	1163002
**No. of base-called reads**	2.34 Gb	1.1 Gb	1.82 Gb
**Read length of N50**	1320	1042	1097
**Mean read quality**	11.6	11.5	11.2
**Mean read length**	1021.1	946	816.6

b

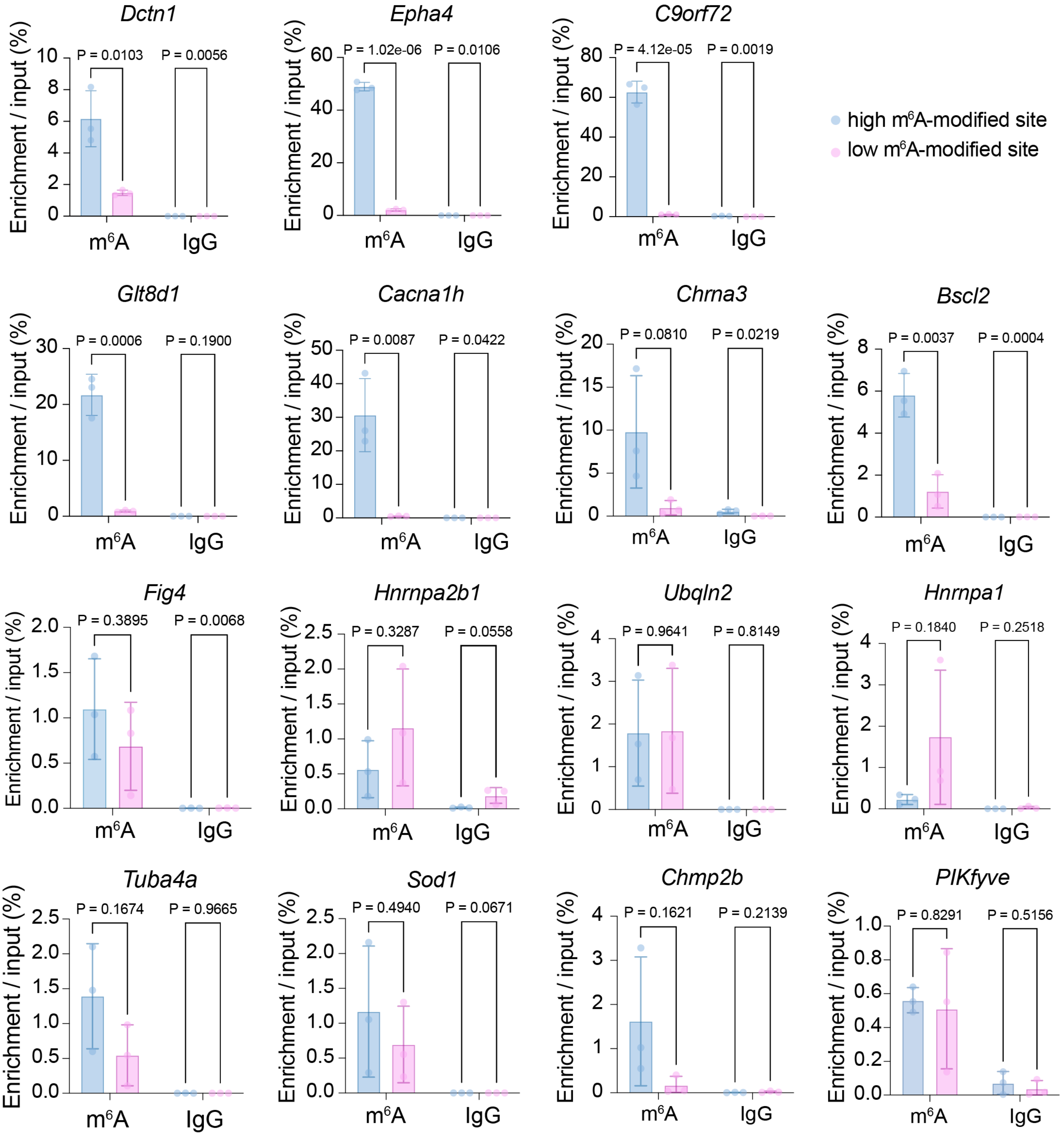

Supplementary Fig. 6: a Summary of the sequencing output and mapping metrics for reads from three mouse ESC-derived MN biological samples. **b** Verification of the predicted ALS risk genes with m^6A-modified sites by m^6A pull-down and IgG pull-down qPCR of selected high m^6A-modified sites and low m^6A-modified sites. Points represent individual biological experiments. All data are presented as mean ± S.D., *n* = 3, with significant P values from two-tailed *t*-tests. Source data are provided as a Source data file.

Supplementary Fig.7

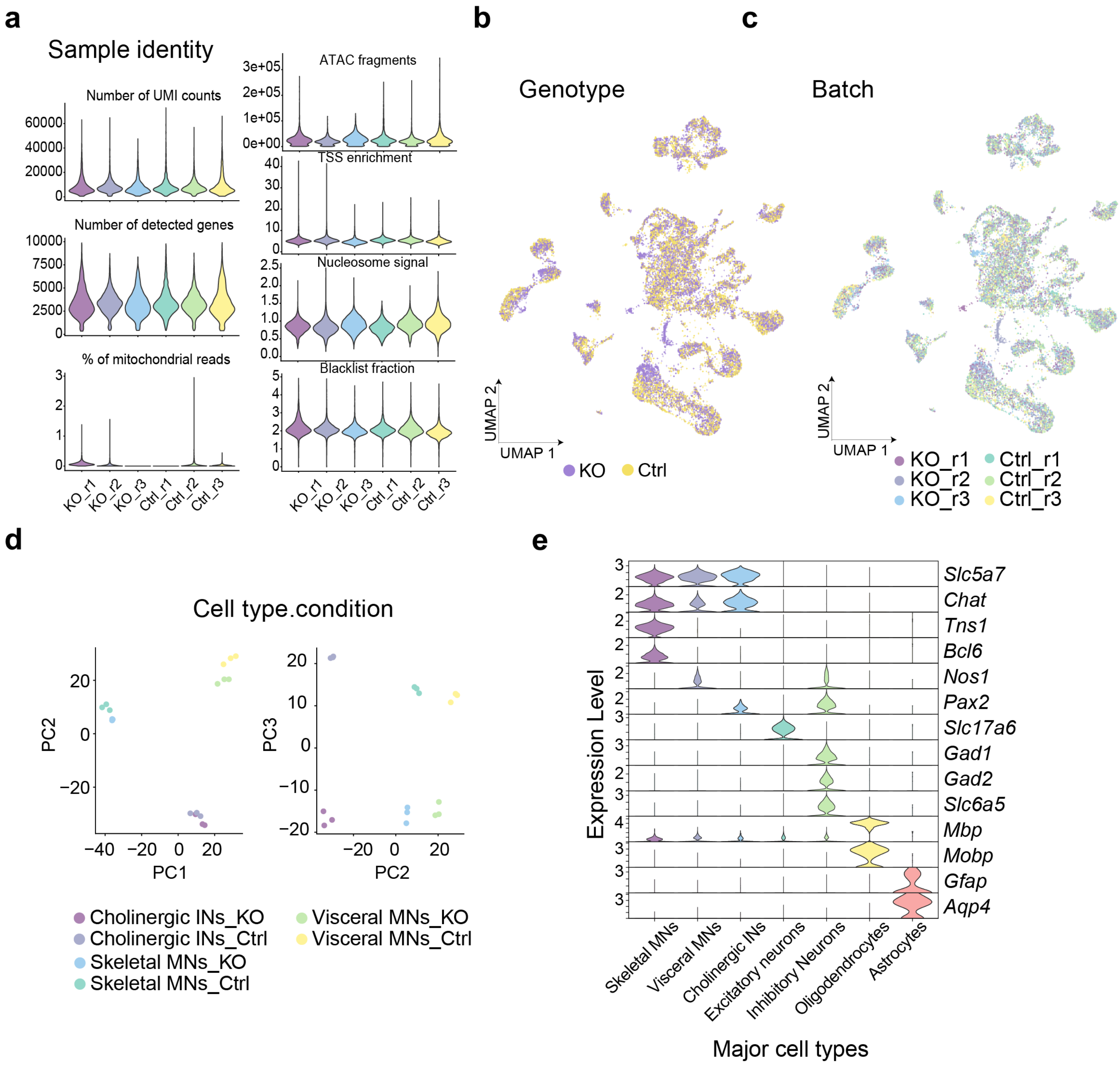

Supplementary Fig. 7: Analysis of the single-nucleus multiome of *Sun1^{sfGFP}; ChAT-Cre; Mettl14^{floxed}* mice. a Violin plots show the UMI and gene counts, percentage of reads mapped to mitochondrial genes, ATAC fragments, TSS enrichment, nucleosome signal, and blacklist fraction of single nuclei collected in our study. All data are analyzed from control (Ctrl) and *Sun1^{sfGFP}; ChAT-Cre; Mettl14^{floxed}* (KO) samples with three biological repeats (r1, r2, and r3). **b** and **c** Integration of all data, including Ctrl and KO samples, reveals no batch effects in our single-nuclei RNA-seq analysis. **d** Principal component analysis (PCA) of the cholinergic neuronal subtype from Ctrl and KO samples. PC1 and PC2 largely segregate samples based on their cell type identity, whereas PC3 distinguishes Ctrl and KO samples, suggesting that *Sun1^{sfGFP}; ChAT-Cre; Mettl14^{floxed}* introduce changes to the transcriptome and chromatin accessibility. **e** Violin plots showing expression patterns of known marker genes (rows) in each cell type (columns).

Supplementary Fig.8

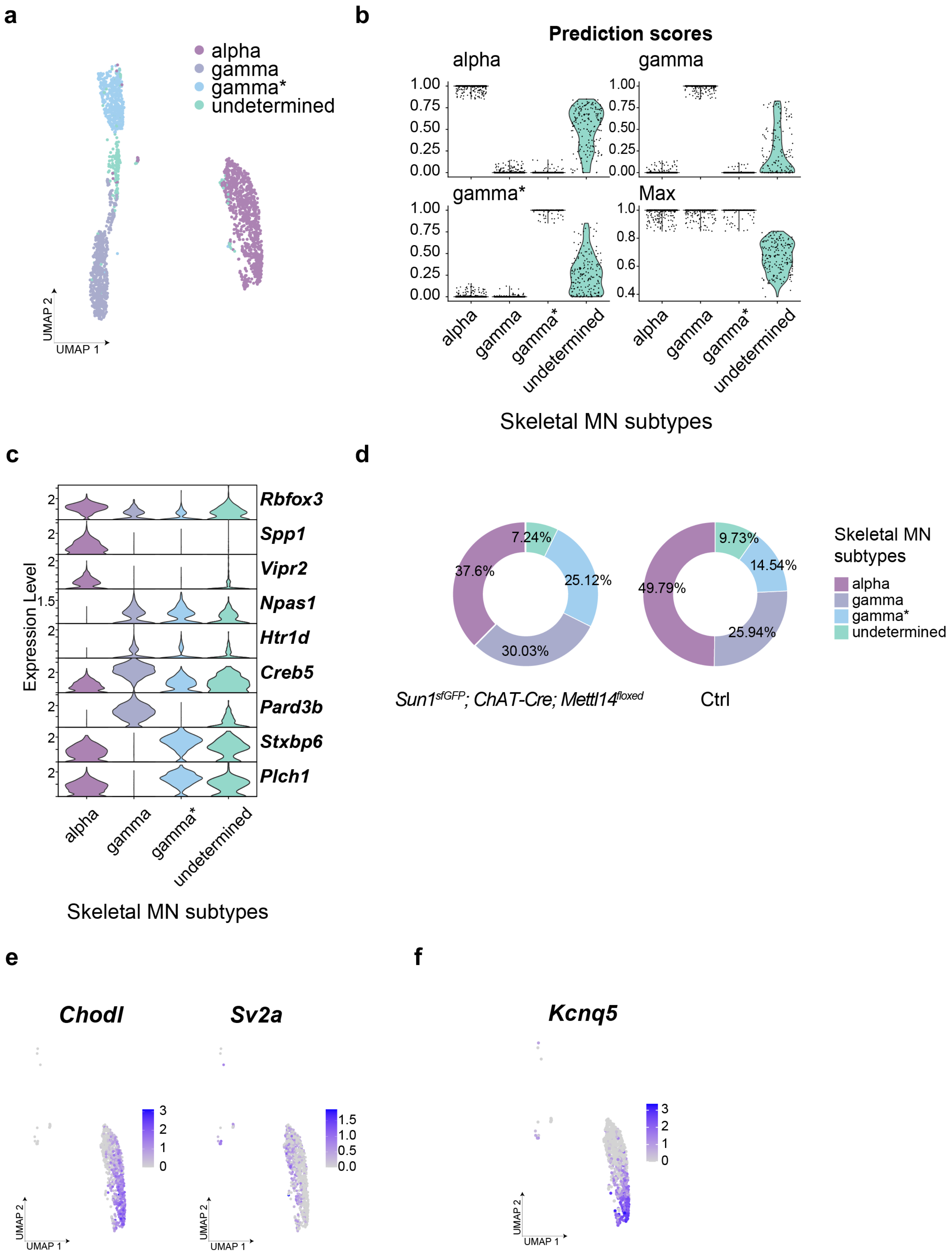

Supplementary Fig. 8: Adult skeletal motor neuron (MN) subtype annotations. a UMAP plots showing skeletal MN subtype heterogeneity. **b** Clusters were annotated based on label transfer prediction from a published single-cell study (Blum et al., 2021). Violin plots show high prediction scores for each subtype, except for undetermined. **c** Violin plots showing expression patterns of known marker genes (rows) in each cluster (columns). **d** Comparison of MN subtype proportions in Ctrl and *Sun1*^{sfGFP}*; ChAT-Cre; Mettl14*^{floxed} samples. **e** and **f** UMAP of known subtype marker genes labels a subset of skeletal MNs. *Chodl* and *Sv2a* label fast and slow α MNs, and *Kcnq5* distinguishes fast-fatigable α MNs.

Supplementary Fig.9

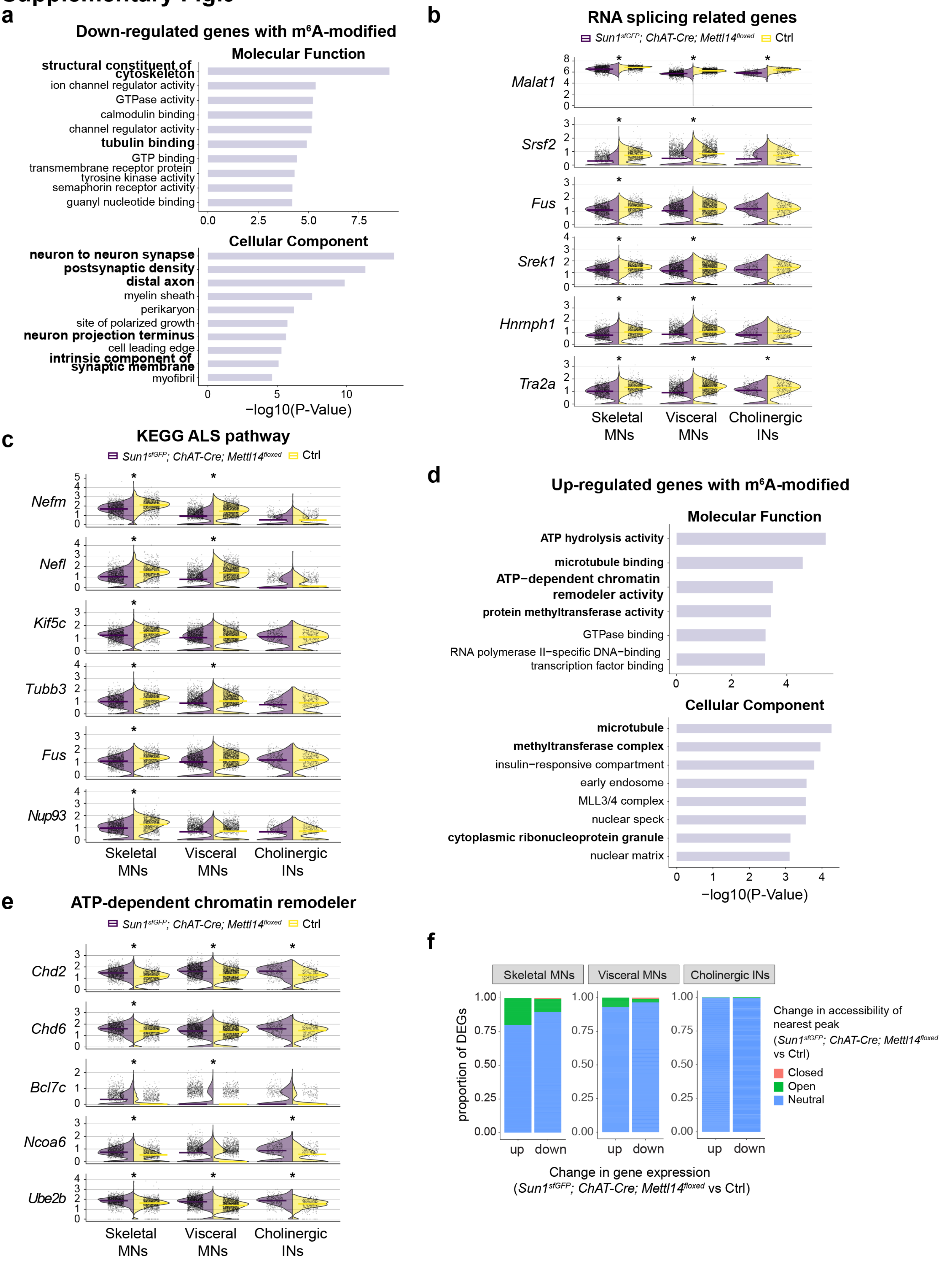

Supplementary Fig. 9: Identification of m^{6}A-modified DEGs in *Sun1^{sfGFP}; ChAT-Cre; Mettl14^{floxed}* mice. **a** and **d** Gene ontology (GO) analysis of the down-regulated (**a**) and up-regulated (**d**) genes in the *Sun1^{sfGFP}; ChAT-Cre; Mettl14^{floxed}* mice that are predicted to be m^{6}A-modified. Terms of interest in this study are highlighted in bold. **b**, **c**, and **e** Violin plots show differential expression of selected genes from each GO term. **f** The proportion of differentially expressed genes with open, closed, or neutral nearby ATAC peaks.

Supplementary Fig.10

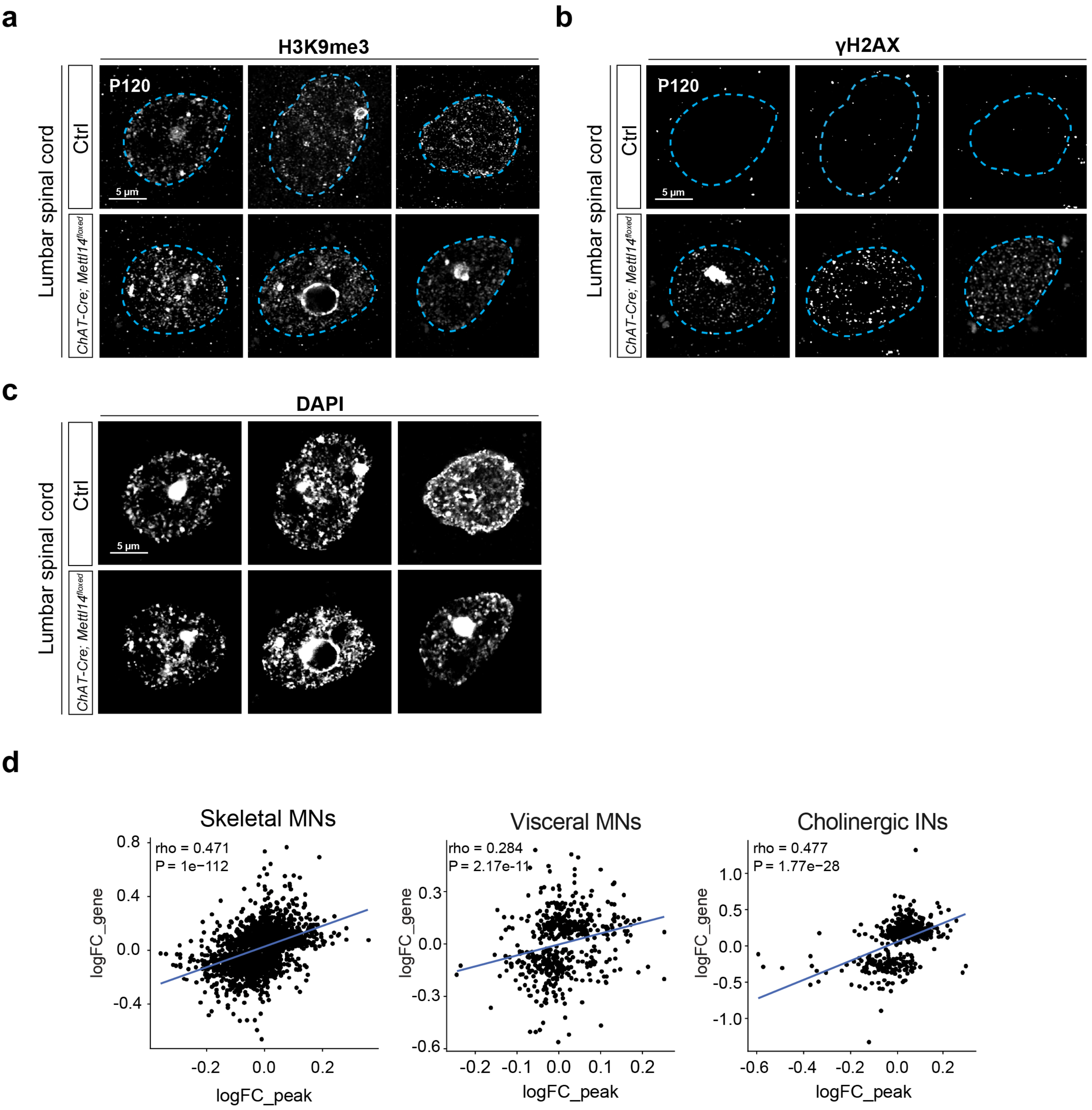

Supplementary Fig. 10: Increased repressive histone modification marks (H3K9me3) and DNA damage response (γH2AX) in *ChAT-Cre; Mettl14^{floxed}* mice. a and **b** Representative images show that all selected MNs display increased signals of H3K9me3 (**a**), γH2AX (**b**), and corresponding DAPI (**c**) in the *ChAT-Cre; Mettl14^{floxed}* mice. (*n* = 3 mice). Scale bars, 5 μm. **d** Spearman's correlation of peak-gene changes in each cholinergic neuronal subtype.

Supplementary Fig.11

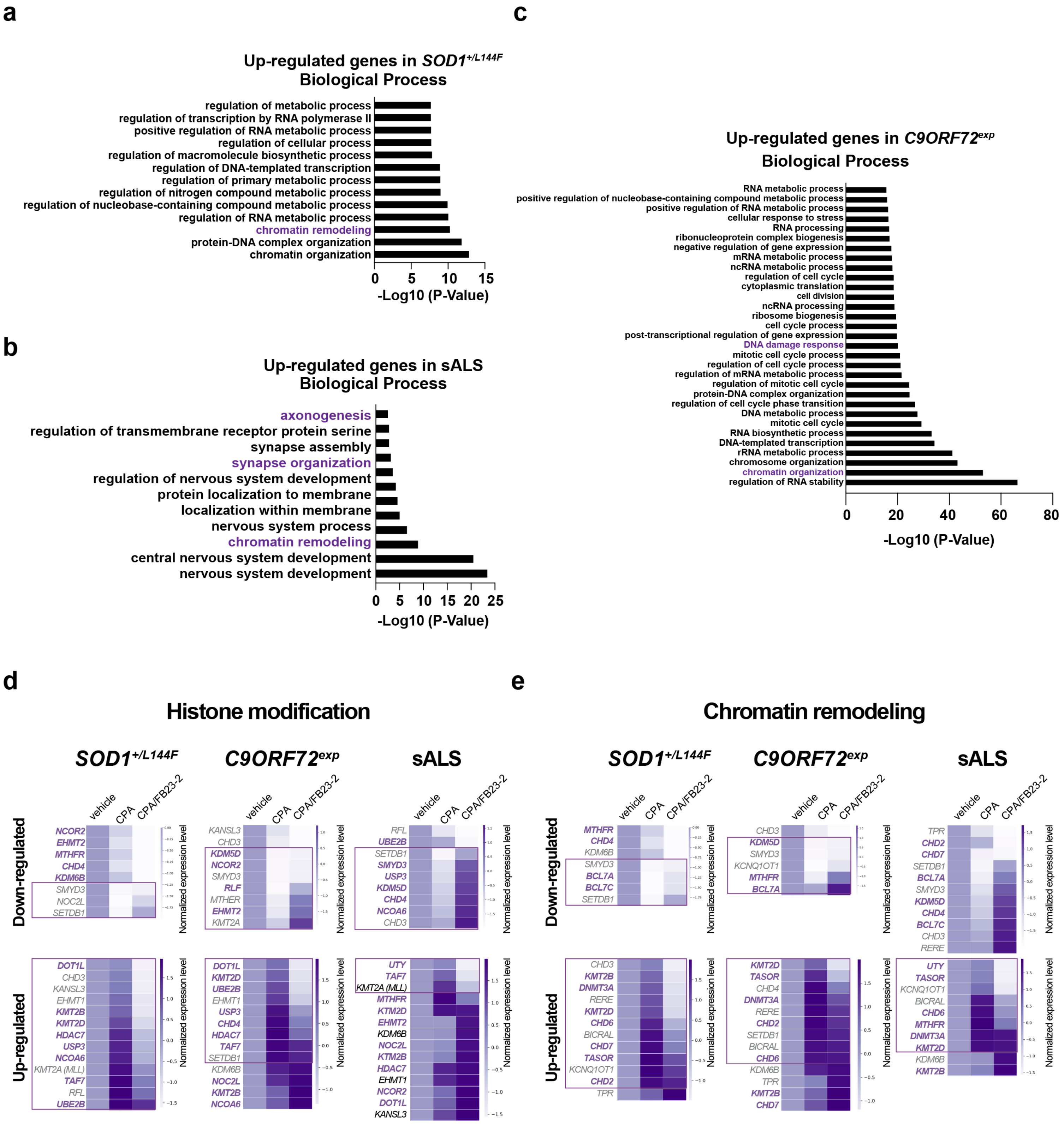

Supplementary Fig. 11: An m^{6}A eraser inhibitor efficiently rescues the histone modification and chromatin remodeling-related genes of human ALS iPSC-derived MNs by restoring dysregulated genes caused by hypo-m^{6}A. a ~ c Gene ontology (GO) analysis of the up-regulated genes in ALS iPSC~MNs revealed chromatin remodeling, DNA damage, synapse organization, and other pathways in the *ChAT-Cre; Mettl14^{floxed}* mice. Terms of interest in this study are highlighted in bold purple. **d** and **e** Heatmaps of normalized expression levels between stress-treated (CPA) ALS-relevant lines with or without subsequent FB23-2 treatment, revealing restoration to control levels (vehicle, highlighted with rectangles) of many histone-related (**d**) and chromatin remodeling-related (**e**) m^{6}A modifications (m^{6}A-modified genes are highlighted in purple and m^{6}A non-modified genes are marked in grey) for the FB23-2-treated groups. A z-score normalization was performed on the normalized read counts across samples for each gene after stress treatment (CPA) with or without subsequent FB23-2 treatment. Samples have been normalized to the vehicle control to reveal the normalized expression level.

Supplementary Fig.12

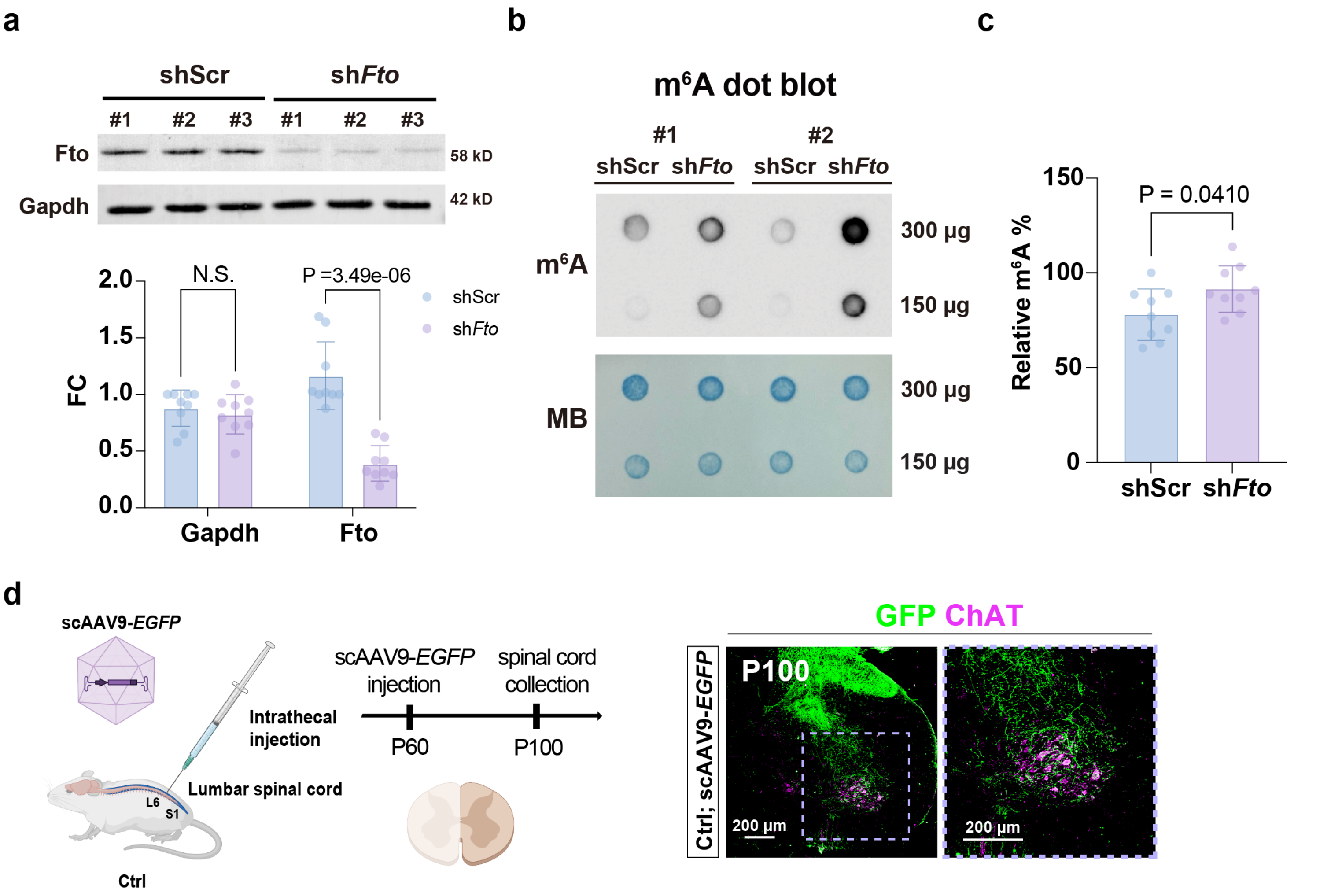

Supplementary Fig. 12: shRNA-mediated Fto inhibition can restore m^{6}A levels. a ~ c Western blots show that all selected *Fto*-shRNAs can efficiently knock down Fto (**a**), with corresponding m^{6}A hypermethylation in C2C12 cells (**b** and **c**). **d** Successful scAAV9-mediated expression was confirmed by detecting GFP in the ChAT^{on} regions in the P100 lumbar spinal cord of a control mouse injected with scAAV9-*EGFP*. Higher magnification of the highlighted area is shown in the panel on the right. Created in BioRender. Chen, J. (2025) https://biorender.com/3m2vxum. Scale bars, 200 μm. All data are presented as mean ± S.D., *n* = 3 mice, with significant P values from two-tailed *t*-tests. N.S., non-significant. Source data are provided as a Source data file.

Supplementary Fig.13

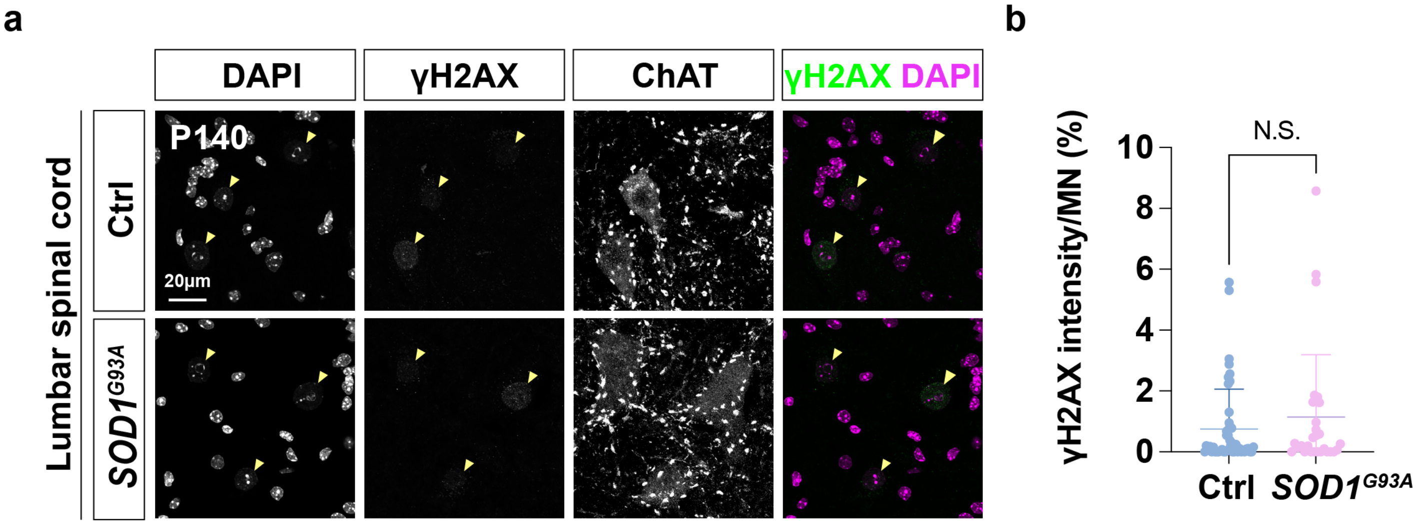

Supplementary Fig. 13: DNA damage marker γH2AX in MNs of *SOD1^{G93A}* mice. a Representative image illustrating γH2AX marks (yellow arrowheads) in the ventral horn of the spinal cord from Ctrl and *SOD1^{G93A}* mice. **b** Quantification of the intensity of lumbar γH2AX^{on} signal (Ctrl: *n* = 6 and *SOD1^{G93A}*: *n* = 4 mice, quantified for all MN nuclei γH2AX^{on} ChAT^{on} double-positive cells; scale bars, 20 µm). Data are presented as mean ± S.D. with significant P values from two-tailed *t*-tests. N.S., non-significant. Source data are provided as a Source data file.

Supplementary Fig.14

a

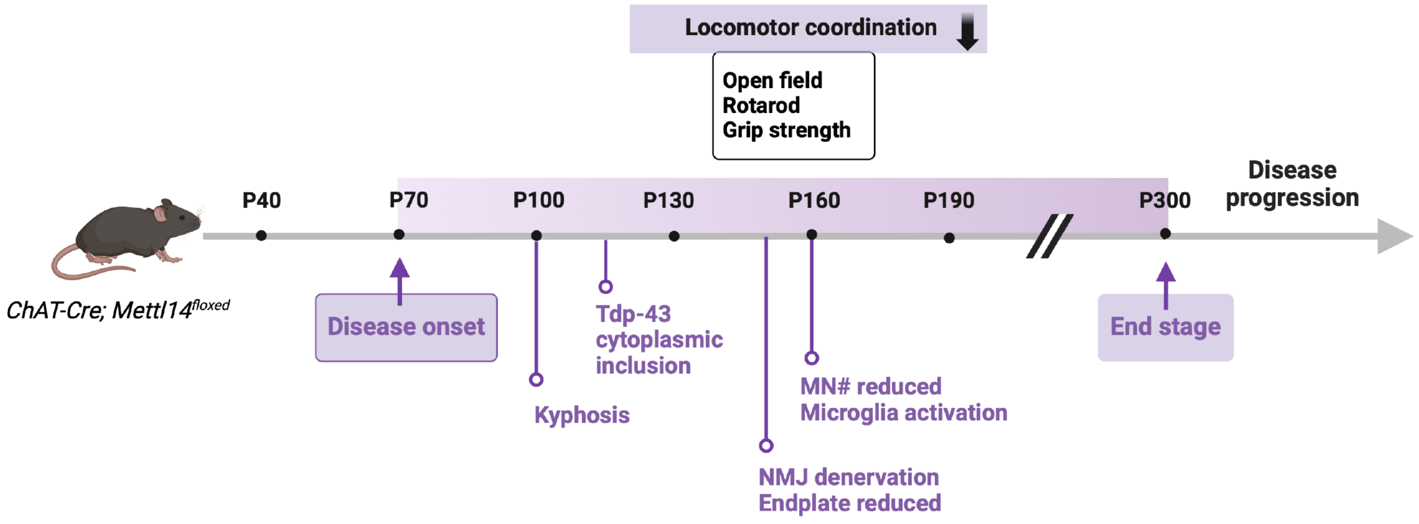

b

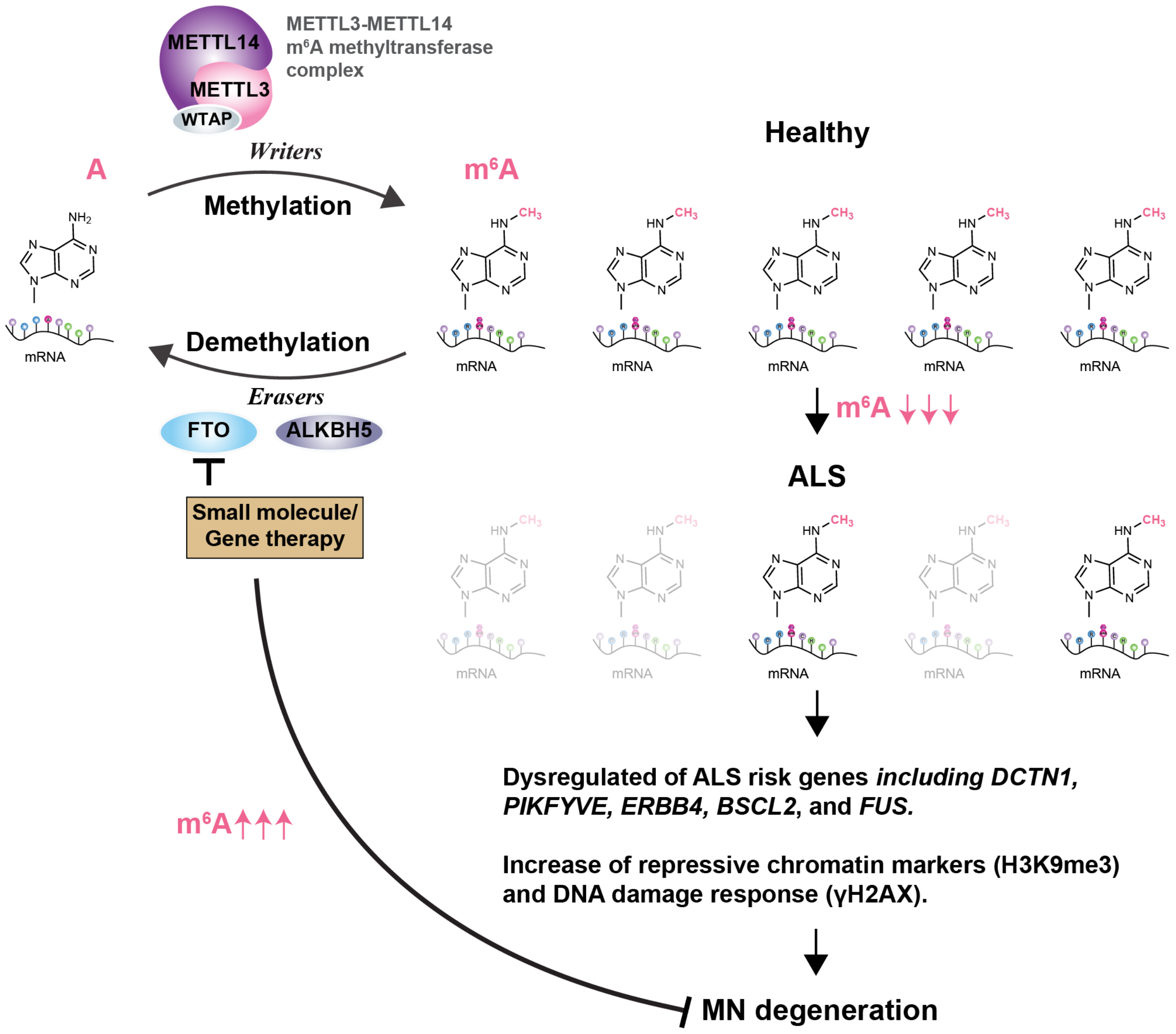

Supplementary Fig. 14: Summary of the phenotypes of *ChAT-Cre; Mettl14^{floxed}* mice and possible mechanism leading to MN degeneration. a Timeline of manifestations of the ALS-like phenotype in the *ChAT-Cre; Mettl14^{floxed}* mice. Created in BioRender. Chen, J. (2025) https://BioRender.com/49vgfn2. **b** A schematic model showing how global m^6A hypomethylation in ALS might lead to MN degeneration and the possible intervention to restore the m^6A reservoir, thereby ameliorating ALS symptoms.

Supplementary Table 1: Reagent or Resource table

**REAGENT or RESOURCE**	**SOURCE**	**IDENTIFIER**
**1st Antibodies**		
Rabbit polyclonal anti-m^{6}A (1:500)	Synaptic Systems	202003; RRID:AB_2279214
Mouse monoclonal anti-m^{6}A (1:100)	Proteintech	68055-1-Ig; RRID:AB_2918796
Mouse monoclonal anti-Neurofilament H, Nonphosphorylated (SMI32) (1:1000)	BioLegend	801701 (clone SMI-32P); RRID:AB_2564642
Goat polyclonal anti-ChAT (1:100)	Millipore	AB144P; RRID:AB_2079751
Rabbit polyclonal anti-Mettl14 (1:1000)	Sigma-Aldrich	HPA038002; RRID:AB_10672401
Rabbit polyclonal anti-Olig2 (1:20000)	Millipore	AB9610; RRID:AB_570666
Guinea pig polyclonal anti-Olig2 (1:100)	Thomas Jessell (Columbia University)	
Rabbit polyclonal anti-Irx3 (1:16000)	Thomas Jessell (Columbia University)	
Mouse monoclonal anti-Nkx2.2 (1:100)	DSHB	74.5A5; RRID:AB_53179
Goat polyclonal anti-Isl1 (1:1000)	Neuromics	GT15051; RRID:AB_2126323
Rabbit anti-Pax6 (1:300)	Covance	PRB-278P; RRID:AB_291612
Mouse monoclonal anti-Neurogenin-2 (1:500)	R&D	MAB3314; RRID:AB_2149520
Guinea pig polyclonal anti-Hb9/Mnx1 (1:1000)	Hynek Wichterle (Columbia University)	
Rabbit polyclonal anti-Lhx3 (1:2000)	Abcam	ab14555; RRID:AB_301332
Rabbit polyclonal anti-Foxp1 (1:20000)	Abcam	ab16645; RRID:AB_732428
Rabbit polyclonal anti-Sox9 (1:2000)	Millipore	AB5535; RRID:AB_2239761
Mouse monoclonal anti-Isl1/2 (1:1000)	DSHB	39.4D5; RRID:AB_2314683
Rabbit polyclonal anti-Iba1 (1:100)	Proteintech	10904-1-AP; RRID:AB_2224377
Rabbit polyclonal anti-TDP-43 (1:100)	Proteintech	10782-2-AP;

		RRID:AB_615042
Mouse monoclonal anti-neurofilament (1:250)	DSHB	#2H3; RRID:AB_531793
Mouse monoclonal anti-SV2 (1:500)	DSHB	#SV2; RRID:AB_2315387
α-Bungarotoxin, Alexa Fluor 555 conjugate (1:500)	Invitrogen	B35451; RRID:AB_2617152
Mouse monoclonal anti-FUS/TLS (1:100)	Santa Cruz	sc-47711 RRID:AB_2105208
Rabbit polyclonal anti-Synapsin I	Sigma-Aldrich	AB1543 RRID: AB_2200400
Rabbit polyclonal anti-Histone H3 (tri-methyl K9) (1:1000)	Abcam	Ab8898; RRID:AB_306848
Mouse monoclonal anti-phospho-Histone H2A.X (Ser139), clone JBW301 (1:1000)	Sigma-Aldrich	05-636; RRID:AB_309864
Rabbit polyclonal anti-phospho-Histone H2A.X (Ser139) (1:500)	Cell signaling	#2577; RRID:AB_2118010
Rabbit polyclonal anti-Fto (1:1000)	Proteintech	27226-1-AP; RRID:AB_2880809
Mouse monoclonal anti-β-Actin (1:4000)	Sigma-Aldrich	A2228; RRID:AB_476697
Rabbit polyclonal anti-Glial Fibrillary Acidic Protein (GFAP) (1:1000)	Millipore	AB5804; RRID:AB_2109645
Mouse monoclonal anti-GAPDH (1:3000)	Millipore	MAB374; RRID:AB2107445
Rabbit polyclonal anti-GFP (1:100)	Invitrogen	A-11122; RRID:AB_221569

**2nd Antibodies**		
Goat polyclonal anti-rabbit IgG-HRP (1:100000)	Santa Cruz	sc-2030; RRID:AB_631747
Goat polyclonal anti-Guinea Pig IgG, Alexa Fluor™ 488 (1:100000)	Invitrogen	A-11073; RRID:AB_2534117
Donkey polyclonal anti-Mouse IgG, Alexa Fluor™ 488 (1:100000)	Invitrogen	A-21202; RRID:AB_141607
Donkey polyclonal anti-Rabbit IgG, Alexa Fluor™ 488 (1:100000)	Invitrogen	A-21206; RRID:AB_2535792
Cy™3 AffiniPure™ Donkey polyclonal anti-Mouse IgG (1:100000)	Jackson ImmunoResearch	715-165-150; RRID:AB_2340813
Cy™3 AffiniPure™ Donkey polyclonal anti-Rabbit IgG (1:100000)	Jackson ImmunoResearch	711-165-152; RRID:AB_2307443
Cy™3 AffiniPure™ Donkey polyclonal anti-Goat IgG (1:100000)	Jackson ImmunoResearch	705-165-147; RRID:AB_2307351

Cy™5 AffiniPure™ Donkey polyclonal Anti-Rabbit IgG (1:100000)	Jackson ImmunoResearch	711-175-152; RRID:AB_2340607
Cy™5 AffiniPure™ Donkey polyclonal anti-Goat IgG (1:100000)	Jackson ImmunoResearch	705-175-147; RRID:AB_2340730
IRDye® 680RD Goat polyclonal anti-Mouse IgG Secondary Antibody (1:10000)	Li-COR	926-68070; RRID:AB_2651128
IRDye® 800CW Goat polyclonal anti-Rabbit IgG Secondary Antibody (1:10000)	Li-COR	926-32211; RRID:AB_2651127

**Critical commercial assays and Kits**		
Chromium Next GEM Single Cell Multiome ATAC + Gene Expression Kit	10x Genomics	1000285
Dynabeads mRNA Purification Kit	Invitrogen	61006
m6A RNA Methylation Assay Kit	Abcam	ab185912
N6-Methyladenosine 5′-monophosphate sodium salt	Sigma-Aldrich	M2780
TruSeq Stranded mRNA library prep kit	Illumina	20020594
IDT for illumina-TruSeq RNA UD Indexes	Illumina	20022371
NextSeq500 HighOutput kit V2.5 (75 cycles)	Illumina	20024906

**Oligonucleotides**		
See Table S5		
**Bacterial and Virus Strains**		
pLKO.1-Scramble (ASN0000000004)	National RNAi Core Facility (Academia Sinica)	N/A
pLKO.1-shMETTL3 (TRC0000034716, TRC0000034717)	National RNAi Core Facility (Academia Sinica)	N/A
pLKO.1-shMETTL14 (TRC0000015936, TRC0000015937)	National RNAi Core Facility (Academia Sinica)	N/A
pscAAV9-CB-H1-GFP	AAV Core Facility (Academia Sinica)	N/A
pscAAV9-H1-shFto-CB-GFP	AAV Core Facility (Academia Sinica)	N/A
pLKO.1-shFto (TRCN0000183897)	National RNAi Core Facility (Academia Sinica)	N/A

Experimental Models: Organisms/Strain		
Mouse: (B6SJL-Tg(SOD1*G93A)1Gur/J	The Jackson Laboratory	RRID: IMSR_JAX:002726
Mouse: *Olig2-Cre*	Tom Jessell (Columbia University)	
Mouse: *Mettl14*^{floxed}		N/A
Mouse: *Olig2-Cre; Mettl14*^{floxed}	This paper	N/A
Mouse: *B6.129S-Chat*$^{tm1(cre)Lowl}$*/MwarJ*	The Jackson Laboratory	RRID: IMSR_JAX:031661
Mouse: *ChAT-Cre; Mettl14*^{floxed}	This paper	N/A
Mouse: B6;129-Gt(ROSA)26Sortm5(CAG-Sun1/sfGFP)Nat/J	The Jackson Laboratory	RRID: IMSR_JAX:021039
Mouse: (B6SJL-Tg(SOD1*G93A)1Gur/J	The Jackson Laboratory	RRID: IMSR_JAX:002726

Experimental Models: Cell Lines		
Mouse: B6 control mouse ESC	This paper	N/A
Human: 29d SOD1$^{+/L144F}$ iPSC line	HSCI iPSC Core, (Boulting et al., 2011)	N/A
Human: 29d corr. SOD1$^{+/+}$ iPSC line	(Tung et al., 2019)	N/A
Human: CS52iALS-C9nxx (C9ORF72exp ~800G4C2) iPSC line	Cedars-Sinai (Answer ALS project).	N/A
Human: CS52iALS-C9n6 ISOxx isogeneic control iPSC line	Cedars-Sinai (Answer ALS project).	N/A
Human: CS47iALS-TDP (*TDP43*^{G298S}) iPSC line	Cedars-Sinai (Answer ALS project).	N/A
Human: sporadic ALS iPSC	Cedars-Sinai (Answer ALS project).	N/A
Human: Health control iPSC line	HSCI iPSC Core	N/A

**Deposited data**		
scRNA+ATACseq data	This paper	GSE290242
Nanopore direct RNA-seq	This paper	GSE290245
ALS iPSC~MNs RNA-seq	This paper	GSE290244
human spinal motor neurons in SOD1 and C9ORF72	GEO	GSE132972 and GSE173115
human postmortem cortex RNA-seq	GEO	GSE122649 and GSE122650
Answer ALS		https://dataportal.answerals.org/home

**Software and Algorithms**		
ArchR	Granja et al.	https://github.com/GreenleafLab/ArchR
CellRanger-ARC	10x Genomics	2.0.1
clusterProfiler	Wu et al.	https://github.com/YuLab-SMU/clusterProfiler
DoubletFinder	McGinnis et al.	https://github.com/chris-mcginnis-ucsf/DoubletFinder
EpiNano	Liu et al.	http://github.com/enovoa/EpiNano
GraphPad Prism version 9.0	GraphPad Software	N/A
HOMER	Heinz et al.	http://homer.ucsd.edu/homer/
Ilatstik	Interactive learning and segmentation toolkit	https://www.ilastik.org
ImageJ Fiji	GraphPad Software	https://imagej.net
m6Anet	Hendra et al.	https://github.com/GoekeLab/m6anet
Macs2	Zhang et al.	https://pypi.org/project/MACS2/
MetaMorph	Molecular Devices	N/A
Presto	Korsunsky et al.	https://github.com/immunogenomics/presto
Seurat	Hao et al.	https://satijalab.org/seurat/
Signac	Stuart et al.	https://stuartlab.org/signac/

Further information and requests for reagents may be directed to, and will be fulfilled by, the Lead Contact, Jun-An Chen (jac2210@gate.sinica.edu.tw) and Ya-Ping Yen (yapingyen@gate.sinica.edu.tw).
