# Supplementary material for: The motor neuron m6A repertoire governs neuronal homeostasis and FTO inhibition mitigates ALS symptom manifestation
Source: Nat Commun. 2025 Apr 30;16:4063. doi: 10.1038/s41467-025-59117-2 (PMC12043976; doi:10.1038/s41467-025-59117-2)
Supplement: Supplementary file 2 — Description of Additional Supplementary Information [file 41467_2025_59117_MOESM2_ESM.docx]

**Description of Additional Supplementary Files**

File Name: Supplementary Data 1

Description: Tab 1-m6 A modifications sites. Tab 2-m6 A modifications genes. (Related to Fig. 5g, 5h, 6e~h, 7a~b, and Supplementary Fig9a, 9d).

File Name: Supplementary Data 2

Description: ALS risk genes with m6 A-modified. (Related to Fig. 5h and Fig. 6g).

File Name: Supplementary Data 3

Description: The predicted m6 A stoichiometry ratio from m6 Anet and Epinano in Tardbp (Tab 1) and Atp13a2 (Tab 2). (Related to Fig. 5i and 5j).

File Name: Supplementary Data 4

Description: Differentially expressed genes (DEGs) within distinct types of cholinergic neurons. P-values are from two-sided bimodal likelihoodratio tests followed by Bonferroni corrections

File Name: Supplementary Data 5

Description: Linked genes of differentially accessible peaks identified from ArchR “peak-to-gene links” analysis in Tab 1: skeletal motor neurons, Tab 2: visceral motor neurons, and Tab 3: cholinergic interneurons. Tab 4: summarizes whether these linked genes are differentially expressed genes (DEGs) and/or m6 A-modified, while Tab 5 summarizes the number of differentially accessible peaks with linked genes in each cell type.

File Name: Supplementary Data 6

Description: The qPCR and cloning primer list were used in this study.

File Name: Supplementary Movie 1

Description: *Olig2-Cre; Mettl14floxed* mice exhibited a shivering

phenotype.

File Name: Supplementary Movie 2

Description: Muscle weakness, one of the major pathological manifestations observed for ALS patients in *ChAT-Cre; Mettl14floxed* mice.

File Name: Supplementary Movie 3

Description: Kinematic analysis revealed that whereas the spinal interneuron circuit remained largely intact, but motor outputs were interrupted in the *ChAT-Cre; Mettl14floxed* mice.

File Name: Supplementary Movie 4

Description: Amelioration of Symptoms in *SOD1G93A*; scAAV9- sh*Fto* mice. Related to Fig.10. The SOD1G93A mouse displayed motor deficit, whereas the *SOD1*G93A; scAAV9-sh*Fto* mouse manifested normal appearance and locomotive ability.
