# Supplementary material for: The motor neuron m6A repertoire governs neuronal homeostasis and FTO inhibition mitigates ALS symptom manifestation
Source: Nat Commun. 2025 Apr 30;16:4063. doi: 10.1038/s41467-025-59117-2 (PMC12043976; doi:10.1038/s41467-025-59117-2)
Supplement: Supplementary file 13 — Reporting Summary [file 41467_2025_59117_MOESM13_ESM.pdf]

nature portfolio

Corresponding author(s): Jun-An Chen, Ya-Ping Yen

Last updated by author(s): 2025.2.13

Reporting Summary

Nature Portfolio wishes to improve the reproducibility of the work that we publish. This form provides structure for consistency and transparency in reporting. For further information on Nature Portfolio policies, see our Editorial Policies and the Editorial Policy Checklist.

Statistics

For all statistical analyses, confirm that the following items are present in the figure legend, table legend, main text, or Methods section.

n/a	Confirmed	
☐	☒	The exact sample size (*n*) for each experimental group/condition, given as a discrete number and unit of measurement
☐	☒	A statement on whether measurements were taken from distinct samples or whether the same sample was measured repeatedly
☐	☒	The statistical test(s) used AND whether they are one- or two-sided<br>*Only common tests should be described solely by name; describe more complex techniques in the Methods section.*
☐	☒	A description of all covariates tested
☐	☒	A description of any assumptions or corrections, such as tests of normality and adjustment for multiple comparisons
☐	☒	A full description of the statistical parameters including central tendency (e.g. means) or other basic estimates (e.g. regression coefficient) AND variation (e.g. standard deviation) or associated estimates of uncertainty (e.g. confidence intervals)
☐	☒	For null hypothesis testing, the test statistic (e.g. *F*, *t*, *r*) with confidence intervals, effect sizes, degrees of freedom and *P* value noted<br>*Give P values as exact values whenever suitable.*
☒	☐	For Bayesian analysis, information on the choice of priors and Markov chain Monte Carlo settings
☒	☐	For hierarchical and complex designs, identification of the appropriate level for tests and full reporting of outcomes
☐	☒	Estimates of effect sizes (e.g. Cohen's *d*, Pearson's *r*), indicating how they were calculated

Our web collection on statistics for biologists contains articles on many of the points above.

Software and code

Policy information about availability of computer code

Data collection	Immunofluorescence results were visualized using LC780 and LC980 microscope. Western blots were visualized using Typhoon. qPCR were performed on the Roche LightCycler® 480 real-time PCR detection system. High-throughput sequencing was performed on the Mi-seq and Next-Seq platform (Illumina).
Data analysis	Cell Ranger pipeline (version 2.1.1, 10x Genomics, RRID:SCR_017344);<br>Prism (version: 10.4.0, GraphPad, RRID:SCR_005375);<br>ZEN (Carl Zeiss, RRID:SCR_013672);<br>Imaris 9.5.1 (version 9.5.1, Bitplane, RRID:SCR_007370);<br>Image J (version 1.51m, NIH, RRID:SCR_003070);<br>MetaXpress (Molecular Devices, RRID: SCR_016654);<br>R and RStudio (Version 2023.09.0+463 (2023.09.0+463); RRID: SCR_001905);<br>Adobe Illustrator 2022 26.0.1 (Adobe, RRID: SCR_010279);<br>Adobe Photoshop 2024.005.20400 (Adobe, RRID:SCR_014199);<br><br>R packages:<br>Seurat (version 4.1.3, Satija et al 2015, RRID:SCR_016341);<br>Signac (version 1.10.0, Stuart et al 2021, RRID:SCR_021158);<br>UMAP (version 0.2.6.0, McInnes, Healy and Melville 2020, https://github.com/lmcinnes/umap);<br>ggplot2 (version 3.5.1, Wickham H 2016; RRID:SCR_014601);

clusterProfiler (version 4.6.2, Yu et al 2012, RRID: SCR_016884)

The codes used for snMultiome sequencing data analysis were an adaptation of standard R packages, as described in the Methods section. The codes and processed data are available on GitHub at https://github.com/jaclab-multiomic/Yen-et-al.-2025-Nat-Comm and figshare at https://figshare.com/projects/Yen-et-al_-2025-Nat-Comm/238025. More detailed information is available upon request.

For manuscripts utilizing custom algorithms or software that are central to the research but not yet described in published literature, software must be made available to editors and reviewers. We strongly encourage code deposition in a community repository (e.g. GitHub). See the Nature Portfolio guidelines for submitting code & software for further information.

Data

Policy information about availability of data

All manuscripts must include a data availability statement. This statement should provide the following information, where applicable:

- Accession codes, unique identifiers, or web links for publicly available datasets
- A description of any restrictions on data availability
- For clinical datasets or third party data, please ensure that the statement adheres to our policy

Data availability
Requests for further information or resources should be directed to and will be filled by the lead authors, Ya-Ping Yen and Jun-An Chen. All the sequencing data generated in this study have been deposited in the Gene Expression Omnibus repository under the GSE accession codes GSE290242, GSE290244, and GSE290245. Previously published human postmortem cortex RNA-seq and human iPSC~MNs are available under the accession codes GSE122649, GSE122650, GSE132972, and GSE173115. Transcriptome data from Answer ALS can be requested via the website: https://www.answerals.org/. All data supporting the findings of this study are available within the paper and Supplementary Information section. Source data are provided in this paper.

Research involving human participants, their data, or biological material

Policy information about studies with human participants or human data. See also policy information about sex, gender (identity/presentation), and sexual orientation and race, ethnicity and racism.

Reporting on sex and gender	This study involved data from human iPSC-derived neuron samples. The information of biological gender regarding the human samples is listed below:<br>1. 29d SOD1+/L144F iPSC line (Female)<br>2. CS4VFTiALS-n1 (male)<br>3. CS47iALS-TDP43 (male)<br>4. CS52iALS-C9n6A (male)<br><br>The influence of sex was not assessed in this study. Rather the effect of CPA treatment was compared between isogenic lines; the FB23-2 treatment to rescue ALS iPSC lines was compared with the vehicle control.
Reporting on race, ethnicity, or other socially relevant groupings	reporting race:<br>1. 29d SOD1+/L144F iPSC line (unknown)<br>2. CS4VFTiALS-n1 (White)<br>3. CS47iALS-TDP43 (unknown)<br>4. CS52iALS-C9n6A (CAUA)
Population characteristics	donor age at sample collection:<br>1. 29d SOD1+/L144F iPSC line (82)<br>2. CS4VFTiALS-n1 (29)<br>3. CS47iALS-TDP43 (64)<br>4. CS52iALS-C9n6A (49+)
Recruitment	No recruitment.
Ethics oversight	The study using patient samples/data was approved by Academia Sinica review boards.(AS-IRB-BM-18042 and AS-IRB-BM-23039)

Note that full information on the approval of the study protocol must also be provided in the manuscript.

Field-specific reporting

Please select the one below that is the best fit for your research. If you are not sure, read the appropriate sections before making your selection.

[x] Life sciences [ ] Behavioural & social sciences [ ] Ecological, evolutionary & environmental sciences

For a reference copy of the document with all sections, see nature.com/documents/nr-reporting-summary-flat.pdf

Life sciences study design

All studies must disclose on these points even when the disclosure is negative.

Sample size	No statistical methods were performed for sample size predetermination, sample size was determined iteratively. We based on previous experiences and reported studies to determine the number of embryos, mice, and cells required for the study. Minimum cluster size in the study was 30 cells in vitro. Minimum size used for the statistic and quantification in the study was three embryos and mice in vivo.
Data exclusions	No data were excluded in mice data.<br>For snMultiome data, cells were retained for subsequent analysis if they displayed a number of genes between 1000 and 5300, UMI counts <30500, as well as <10% mitochondrial counts. During initial clustering, non-motor neurons were filtered based on expression of known markers, retaining only our cells of interest, as detailed in the methods in the manuscript.
Replication	All attempts at replication were successful and can be performed independently.
Randomization	No randomization was performed. We plated the cells in random positions in multi-well plates and randomly assigned them to different experiment groups. We randomly took fluorescence images under the microscope.
Blinding	Blinding was conducted solely during quantification comparisons between control and mutant mice. Moreover, blinding was inconsequential for high throughput sequencing as libraries were pooled prior to loading.

Reporting for specific materials, systems and methods

We require information from authors about some types of materials, experimental systems and methods used in many studies. Here, indicate whether each material, system or method listed is relevant to your study. If you are not sure if a list item applies to your research, read the appropriate section before selecting a response.

Materials & experimental systems

n/a	Involved in the study	
☐	☒	Antibodies
☐	☒	Eukaryotic cell lines
☒	☐	Palaeontology and archaeology
☐	☒	Animals and other organisms
☒	☐	Clinical data
☒	☐	Dual use research of concern
☒	☐	Plants

Methods

n/a	Involved in the study	
☒	☐	ChIP-seq
☒	☐	Flow cytometry
☒	☐	MRI-based neuroimaging

Antibodies

Antibodies used

Primary antibodies:
Rabbit polyclonal anti-m6A (1:500) Synaptic Systems 202003; RRID:AB_2279214
Mouse monoclonal anti-m6A (1:100) Proteintech 68055-1-Ig; RRID:AB_2918796
Rabbit monoclonal anti-β3-Tubulin (D71G9) (1:100) Cell signaling #5568; RRID:AB_10694505
Mouse monoclonal anti-NeuN (RBFOX3) (1:2000) Sigma-Aldrich MAB377; RRID:AB_2298772
Mouse monoclonal anti-Neurofilament H, Nonphosphorylated (SMI32) (1:1000) BioLegend 801701 (clone SMI-32P); RRID:AB_2564642
Rabbit polyclonal anti-Synapsin I (1:1000) Sigma-Aldrich ab1543;RRID:AB 2200400
Rabbit polyclonal anti-Foxp1 (1:20000) Abcam ab16645; RRID:AB_732428
Goat polyclonal anti-Isl1 (1:1000) Neuromics GT15051; RRID:AB_2126323
Goat polyclonal anti-ChAT (1:100) Millipore AB144P; RRID:AB_2079751
Rabbit polyclonal anti-Iba1 (1:100) Proteintech 22309-1-AP; RRID:AB_2224377
Mouse monoclonal anti-neurofilament (1:250) DSHB #2H3; RRID:AB_531793
Mouse monoclonal anti-SV2 (1:500) DSHB #SV2; RRID:AB_2315387
α-Bungarotoxin, Alexa Fluor 555 conjugate (1:500) Invitrogen B35451; RRID:AB_2617152
Rabbit polyclonal anti-TDP-43 (1:100) Proteintech 10782-2-AP; RRID:AB_615042
Rabbit polyclonal anti-Mettl14 (1:1000) Sigma-Aldrich HPA038002; RRID:AB_10672401
Rabbit polyclonal anti-Olig2 (1:20000) Millipore AB9610; RRID:AB_570666
Guinea pig polyclonal anti-Olig2 (1:100) Thomas Jessell (Columbia University)
Rabbit polyclonal anti-Irx3 (1:16000) Thomas Jessell (Columbia University)
Mouse monoclonal anti-Nkx2.2 (1:100) DSHB 74.5A5; RRID:AB_53179
Rabbit anti-Pax6 (1:300) Covance PRB-278P; RRID:AB_291612
Mouse monoclonal anti-Neurogenin-2 (1:500) R&D MAB3314; RRID:AB_2149520
Guinea pig polyclonal anti-Hb9/Mnx1 (1:1000) Hynek Wichterle (Columbia University)
Rabbit polyclonal anti-Lhx3 (1:2000) Abcam ab14555; RRID:AB_301332

Mouse monoclonal anti-Isl1/2 (1:1000) DSHB 39.4D5; RRID:AB_2314683
Rabbit polyclonal anti-Sox9 (1:2000) Millipore AB5535; RRID:AB_2239761
Rabbit polyclonal anti-Histone H3 (tri-methyl K9) (1:1000) Abcam Ab8898; RRID:AB_306848
Mouse monoclonal anti-phospho-Histone H2A.X (Ser139), clone JBW301 (1:1000) Sigma-Aldrich 05-636; RRID:AB_309864
Rabbit polyclonal anti-phospho-Histone H2A.X (Ser139) (1:500) Cell signaling #2577; RRID:AB_2118010
Rabbit polyclonal anti-Fto (1:1000) Proteintech 27226-1-AP; RRID:AB_2880809
Rabbit polyclonal anti-Glial Fibrillary Acidic Protein (GFAP) (1:1000) Millipore AB5804; RRID:AB_2109645
Rabbit polyclonal anti-GFP (1:100) Invitrogen A-11122; RRID:AB_221569
Mouse monoclonal anti-β-Actin (1:4000) Sigma-Aldrich A2228; RRID:AB_476697
Mouse monoclonal anti-GAPDH (1:3000) Millipore MAB374; RRID:AB_2107445

Secondary antibodies:
Goat polyclonal anti-rabbit IgG-HRP (1:100000) Santa Cruz sc-2030; RRID:AB_631747
Goat polyclonal anti-Guinea Pig IgG, Alexa Fluor™ 488 (1:100000) Invitrogen A-11073; RRID:AB_2534117
Donkey polyclonal anti-Mouse IgG, Alexa Fluor™ 488 (1:100000) Invitrogen A-21202; RRID:AB_141607
Donkey polyclonal anti-Rabbit IgG, Alexa Fluor™ 488 (1:100000) Invitrogen A-21206; RRID:AB_2535792
Cy™3 AffiniPure™ Donkey polyclonal anti-Mouse IgG (1:100000) Jackson ImmunoResearch 715-165-150; RRID:AB_2340813
Cy™3 AffiniPure™ Donkey polyclonal anti-Rabbit IgG (1:100000) Jackson ImmunoResearch 711-165-152; RRID:AB_2307443
Cy™3 AffiniPure™ Donkey polyclonal anti-Goat IgG (1:100000) Jackson ImmunoResearch 705-165-147; RRID:AB_2307351
Cy™5 AffiniPure™ Donkey polyclonal Anti-Rabbit IgG (1:100000) Jackson ImmunoResearch 711-175-152; RRID:AB_2340607
Cy™5 AffiniPure™ Donkey polyclonal anti-Goat IgG (1:100000) Jackson ImmunoResearch 705-175-147; RRID:AB_2340730
IRDye® 680RD Goat polyclonal anti-Mouse IgG Secondary Antibody (1:10000) Li-COR 925-68070; RRID:AB_2651128
IRDye® 800CW Goat polyclonal anti-Rabbit IgG Secondary Antibody (1:10000) Li-COR 925-32211; RRID:AB_2651127

Validation

All commercially acquired antibodies used in this study were thoroughly validated by manufacturer and published studies. Individual antibody profiles and relevant citations were provided in the links.

Rabbit polyclonal anti-m6A (https://sysy.com/product/202003)
Mouse monoclonal anti-m6A (https://www.ptglab.com/products/m6A-Antibody-68055-1-Ig.htm)
Rabbit monoclonal anti-β3-Tubulin (https://www.cellsignal.com/products/primary-antibodies/b3-tubulin-d71g9-xp-rabbit-mab/5568)
Mouse monoclonal anti-NeuN (RBFOX3) (https://www.sigmaaldrich.com/TW/en/product/mm/mab377)
Mouse monoclonal anti-Neurofilament H, Nonphosphorylated (SMI32) (https://www.biolegend.com/fr-fr/products/purified-anti-neurofilament-h-nf-h-nonphosphorylated-antibody-11475?GroupID=BLG15643)
Rabbit polyclonal anti-Synapsin(https://www.merckmillipore.com/TW/zh/product/Synapsin-I-Antibody,MM_NF-AB1543)
Rabbit polyclonal anti-Foxp1 (https://www.abcam.com/products/primary-antibodies/foxp1-antibody-ab16645.html)
Goat polyclonal anti-Isl1 (https://www.antibodyregistry.org/AB_2126323)
Goat polyclonal anti-ChAT (https://www.merckmillipore.com/TW/zh/product/Anti-Choline-Acetyltransferase-Antibody,MM_NF-AB144P)
Rabbit polyclonal anti-Iba1 (https://www.ptglab.com/products/IBA1-Antibody-10904-1-AP.htm)
Mouse monoclonal anti-neurofilament (https://dshb.biology.uiowa.edu/2H3)
Mouse monoclonal anti-SV2 (https://dshb.biology.uiowa.edu/SV2)
α-Bungarotoxin, Alexa Fluor 555 conjugate (https://www.thermofisher.com/order/catalog/product/B35451?SID=srch-hj-B35451)
Rabbit polyclonal anti-TDP-43 (https://www.ptglab.com/Products/TARDBP-Antibody-10782-2-AP.htm)
Rabbit polyclonal anti-Mettl14 (https://www.sigmaaldrich.com/TW/en/product/sigma/hpa038002)
Rabbit polyclonal anti-Olig2 (https://www.merckmillipore.com/TW/zh/product/Anti-Olig-2-Antibody,MM_NF-AB9610)
Mouse monoclonal anti-Nkx2.2 (https://dshb.biology.uiowa.edu/74-5A5)
Rabbit anti-Pax6 (https://www.antibodyregistry.org/AB_291612)
Mouse monoclonal anti-Neurogenin-2 (https://www.rndsystems.com/products/human-rat-neurogenin-2-antibody-7g4_mab3314)
Rabbit polyclonal anti-Lhx3 (https://www.antibodyregistry.org/AB_301332)
Mouse monoclonal anti-Isl1/2 (https://dshb.biology.uiowa.edu/39-4D5)
Rabbit polyclonal anti-Sox9 (https://www.sigmaaldrich.com/TW/en/product/sigma/zrb5535?utm_source=google&utm_medium=cpc&utm_campaign=8809292847&utm_content=91641849027&gclid=Cj0KCQjwqdqvBhCPARIsANrmZhOxXPHi_51wO2Iftp9wbCbGOrCV-Qyvb6XrfgegngW2R6DFf4kGKwIaAuCEEALw_wcB)
Rabbit polyclonal anti-Histone H3 (https://www.abcam.com/products/primary-antibodies/histone-h3-tri-methyl-k9-antibody-chip-grade-ab8898.html)
Mouse monoclonal anti-phospho-Histone H2A.X (Ser139), clone JBW301 (https://www.sigmaaldrich.com/TW/en/product/sigma/ZMS05636)
Rabbit polyclonal anti-phospho-Histone H2A.X (Ser139) (https://www.cellsignal.com/products/primary-antibodies/phospho-histone-h2a-x-ser139-antibody/2577)
Rabbit polyclonal anti-Fto (https://www.ptglab.com/Products/FTO-Antibody-27226-1-AP.htm)
Rabbit polyclonal anti-Glial Fibrillary Acidic Protein (GFAP) (https://www.merckmillipore.com/TW/zh/product/Anti-Glial-Fibrillary-Acidic-Protein-GFAP-Antibody,MM_NF-AB5804)
Mouse monoclonal anti-β-Actin (https://www.sigmaaldrich.com/TW/en/substance/monoclonalantibactinantibodyproducedinmouse1234598765)
Mouse monoclonal anti-GAPDH (https://www.sigmaaldrich.com/TW/en/product/mm/mab374)
Goat polyclonal anti-rabbit IgG-HRP (https://www.antibodyregistry.org/AB_291612)
Goat polyclonal anti-Guinea Pig IgG, Alexa Fluor™ 488 (https://www.thermofisher.com/antibody/product/Goat-anti-Guinea-Pig-IgG-

H-L-Highly-Cross-Adsorbed-Secondary-Antibody-Polyclonal/A-11073)
Donkey polyclonal anti-Mouse IgG, Alexa Fluor™ 488 (https://www.thermofisher.com/antibody/product/Donkey-anti-Mouse-IgG-H-L-Highly-Cross-Adsorbed-Secondary-Antibody-Polyclonal/A-21202)
Donkey polyclonal anti-Rabbit IgG, Alexa Fluor™ 488 (https://www.thermofisher.com/antibody/product/Donkey-anti-Rabbit-IgG-H-L-Highly-Cross-Adsorbed-Secondary-Antibody-Polyclonal/A-21206)
Cy™3 AffiniPure™ Donkey polyclonal anti-Mouse IgG (https://www.jacksonimmuno.com/catalog/products/715-165-150)
Cy™3 AffiniPure™ Donkey polyclonal anti-Rabbit IgG (https://www.jacksonimmuno.com/catalog/products/711-165-152)
Cy™3 AffiniPure™ Donkey polyclonal anti-Goat IgG (https://www.jacksonimmuno.com/catalog/products/705-165-147)
Cy™5 AffiniPure™ Donkey polyclonal Anti-Rabbit IgG (https://www.jacksonimmuno.com/catalog/products/711-175-152)
Cy™5 AffiniPure™ Donkey polyclonal anti-Goat IgG (https://www.jacksonimmuno.com/catalog/products/705-175-147)
IRDye® 680RD Goat polyclonal anti-Mouse IgG Secondary Antibody (https://www.licor.com/bio/reagents/irdye-680rd-goat-anti-mouse-igg-secondary-antibody)
IRDye® 800CW Goat polyclonal anti-Rabbit IgG Secondary Antibody (https://www.licor.com/bio/reagents/irdye-800cw-goat-anti-rabbit-igg-secondary-antibody)

information about antibody gifts:
Guinea pig polyclonal anti-Olig2 (1:100) Thomas Jessell (Columbia University)
Rabbit polyclonal anti-Irx3 (1:16000) Thomas Jessell (Columbia University)
Guinea pig polyclonal anti-Hb9/Mnx1 (1:1000) Hynek Wichterle (Columbia University)

Eukaryotic cell lines

Policy information about cell lines and Sex and Gender in Research

Cell line source(s)
29d SOD1+/L144F iPSC line was obtain from HSCI iPS Core. CS4VFTiALS-n1, CS47iALS-TDP43, CS52iALS-C9n6A and their isogenetic control iPSC lines were obtain from Cedars-Sinai

Authentication
All iPSC lines were authenticated by the expression of neuron marker (immunostaining and qPCR) after motor neuron differentiation.

Mycoplasma contamination
No mycoplasma contamination was detected in all iPSC lines.

Commonly misidentified lines
(See ICLAC register)
No commonly misidentified lines were used

Animals and other research organisms

Policy information about studies involving animals; ARRIVE guidelines recommended for reporting animal research, and Sex and Gender in Research

Laboratory animals
Mus musculus (B6SJL-Tg(SOD1*G93A)1Gur/J (The Jackson Laboratory; RRID: IMSR_JAX:002726)
Mus musculus Olig2-Cre (Tom Jessell, Columbia University)
Mus musculus Mettl14floxed (Chuan-He, University of Chicago)
Mus musculus B6.129S-Chattm1(cre)Lowl/MwarJ (The Jackson Laboratory, RRID: IMSR_JAX:031661)
Mus musculus B6;129-Gt(ROSA)26Sortm5(CAG-Sun1/sfGFP)Nat/J (The Jackson Laboratory RRID: IMSR_JAX:021039)
Mus musculus B6SJL-Tg(SOD1*G93A)1Gur/J (The Jackson Laboratory RRID: IMSR_JAX:002726)
B6;129-Gt(ROSA)26Sortm5(CAG-Sun1/sfGFP)Nat/J

Related to Fig.4b,c. (n mice at P40, P70, P100, P130, and P160 from males/females)
Ctrl: n = 3/3, 8/9, 5/10, 11/10, and 10/10
ChAT-Cre; Mettl14floxed: n = 3/3, 7/11, 7/15, 9/12, and 8/10
Related to Fig.4d. (n mice at P40, P70, P100, P130, and P160 from males/females)
Ctrl: n = 3/6, 12/12, 12/14, 12/10, and 6/10
ChAT-Cre; Mettl14floxed: n = 3/6, 10/14, 10/17, 812, and 7/9 at P40, P70, P100, P130
Related to Fig.4f. (n mice at P40, P70, P100, P130, and P160 from males/females)
Ctrl: n = 3/3, 7/9, 6/8, 8/9, and 8/9
ChAT-Cre; Mettl14floxed: n = 3/3, 6/11, 6/11, 7/12, and 7/9
Related to Fig.4h:n = 6 (3 from P180 and 3 from > P210 mice).

Related to Fig.9j. (n mice of male/female)
Ctrl: 5/1, 4/3, 3/3, 4/3,
Ctrl;scAAV9-shFto: 5/1, 5/2, 5/2, 5/2
SOD1G93A : 2/3, 5/6, 4/5, 4/3
SOD1G93A;scAAV9-shFto: 3/2, 5/3, 4/3, 4/3
Related to Fig.9k (n mice of male/female)
Ctrl: 6/2, 4/3, 4/3, 4/3
Ctrl;scAAV9-shFto: 5/1, 5/2, 5/2, 5/2

	SOD1G93A : 3/4, 5/6, 5/6, 5/5<br>SOD1G93A;scAAV9-shFto: 5/2, 5/3, 5/3, 5/3<br>Related to Fig.9l(n mice of male/female)<br>Ctrl: 6/2, 4/3, 4/3, 4/1<br>Ctrl;scAAV9-shFto: 5/1, 5/2, 5/2, 5/2<br>SOD1G93A : 4/4, 5/6, 5/6, 4/3<br>SOD1G93A;scAAV9-shFto: 5/2, 5/3, 5/3, 5/3
Wild animals	No wild animals were used in this study.<br>All animals were compare with the litermate controls.
Reporting on sex	no apply for one sex in this study
Field-collected samples	No field-collected samples were used in this study.
Ethics oversight	All mouse experiments conform to guidelines approved and overseen by Academia Sinica Institutional Animal Care and Use Committee. (IACUC no. 13-06-559)

Note that full information on the approval of the study protocol must also be provided in the manuscript.

Plants

Seed stocks	n/a
Novel plant genotypes	n/a
Authentication	n/a
